# Supplementary material for: Reasons for Treatment Discontinuation and Their Effect on Outcomes of Immunotherapy in Southwest Finland: A Retrospective, Real-World Cohort Study
Source: Cancers (Basel). 2024 Feb 7;16(4):709. doi: 10.3390/cancers16040709 (PMC10887274; doi:10.3390/cancers16040709)
Supplement: Supplementary file 1 [file cancers-16-00709-s001.zip › Figures S1 and S2.pdf]

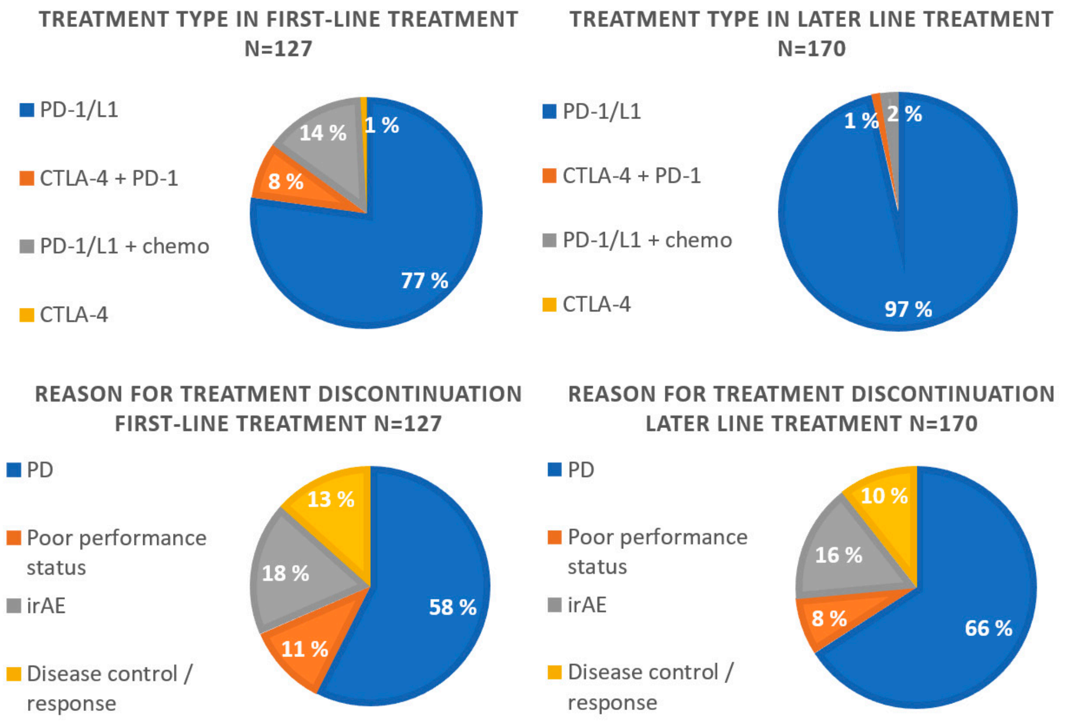

Figure S1. Treatment regimens and the reasons for treatment discontinuation according to treatment line.

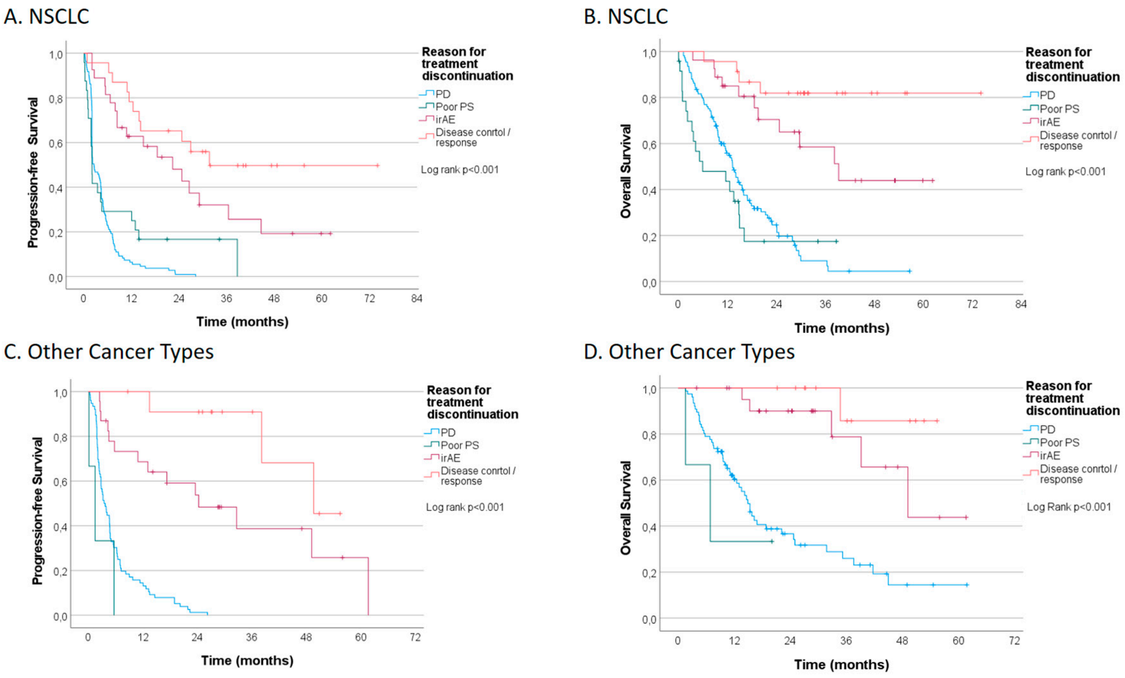

Figure S2. The effect of the reason for treatment discontinuation on progression-free and overall survival in patients with NSCLC (panels (A,B)) and in patients with other cancer types (panels (C,D)).
