# Supplementary material for: Reasons for Treatment Discontinuation and Their Effect on Outcomes of Immunotherapy in Southwest Finland: A Retrospective, Real-World Cohort Study
Source: Cancers (Basel). 2024 Feb 7;16(4):709. doi: 10.3390/cancers16040709 (PMC10887274; doi:10.3390/cancers16040709)
Supplement: Supplementary file 1 [file cancers-16-00709-s001.zip › Table S1 (clean) 25JAN2024.pdf]

Table S1. Cancer types treated with immune checkpoint inhibitors in routine clinical practice in Southwest Finland

Cancer type	Number of patients with percentage
Non-small cell lung cancer	187 (59.0%)
Cutaneous melanoma	50 (15.8%)
Renal cell carcinoma	30 (9.5%)
Urothelial cancer	12 (3.8%)
Gastroenterological cancers	8 (2.5%)
Head and neck cancer	8 (2.5%)
Mesothelioma	6 (1.9%)
Mucosal melanoma	4 (1.3%)
Cutaneous squamous cell carcinoma	3 (0.9%)
Gynecological cancers	2 (0.6%)
Lymphoma	2 (0.6%)
Breast cancer	2 (0.6%)
Primary central nervous system melanoma	1 (0.3%)
Uveal melanoma	1 (0.3%)
Adrenal carcinoma	1 (0.3%)
All	317 (100%)
