# Supplementary material for: Reasons for Treatment Discontinuation and Their Effect on Outcomes of Immunotherapy in Southwest Finland: A Retrospective, Real-World Cohort Study
Source: Cancers (Basel). 2024 Feb 7;16(4):709. doi: 10.3390/cancers16040709 (PMC10887274; doi:10.3390/cancers16040709)
Supplement: Supplementary file 1 [file cancers-16-00709-s001.zip › Table S2 (clean) 25JAN2024.pdf]

Table S2. Immune related adverse events (irAEs) leading to treatment discontinuation

irAE	Number of patients with percentage	Number of patients with grade according to CTCAE version 5.0
Pneumonitis	13 (4.1%)	8 Intolerable / recurrent Gr 2<br>4 Gr 3<br>1 Gr 4
Hepatitis	11 (3.4%)	3 Intolerable / recurrent Gr 2<br>7 Gr 3<br>1 Gr 4
Colitis	10 (3.1%)	2 Intolerable / recurrent Gr 2<br>7 Gr 3<br>1 Gr 4
Hyperthyroidism	3 (0.9%)	3 Intolerable / recurrent Gr 2
Pancreatitis	2 (0.6%)	2 Gr 3
Myocarditis/myositis	2 (0.6%)	2 Gr 3
Dermatitis	2 (0.6%)	2 Gr 3
Arthritis	2 (0.6%)	1 Intolerable / recurrent Gr 2<br>1 Gr 3
Encephalitis	1 (0.3%)	1 Gr 5
Delirium	1 (0.3%)	1 Gr 3
Peripheral neuropathy	1 (0.3%)	1 Intolerable / recurrent Gr 2
Acute onset type 1 diabetes mellitus	1 (0.3%)	1 Gr 4
Renal tubular acidosis	1 (0.3%)	1 Gr 4

Gr=grade
